# Association of MRI findings and expert diagnosis of symptomatic meniscal tear among middle-aged and older adults with knee pain

**DOI:** 10.1186/s12891-016-1010-2

**Published:** 2016-04-11

**Authors:** Bhushan R. Deshpande, Elena Losina, Savannah R. Smith, Scott D. Martin, R. John Wright, Jeffrey N. Katz

**Affiliations:** Department of Orthopaedic Surgery, Orthopaedic and Arthritis Center for Outcomes Research, Brigham and Women’s Hospital, 75 Francis Street, BC-4-016, Boston, MA 02115 USA; Division of Rheumatology, Section of Clinical Sciences, Immunology and Allergy, Brigham and Women’s Hospital, Boston, MA USA; Harvard Medical School, Boston, MA USA; Department of Biostatistics, Boston University School of Public Health, Boston, MA USA; Department of Orthopaedic Surgery, Brigham and Women’s Hospital, Boston, MA USA; Departments of Epidemiology and Environmental Health, Harvard T. H. Chan School of Public Health, Boston, MA USA

**Keywords:** Knee pain, Meniscal tear, MRI, Diagnosis

## Abstract

**Background:**

Our aim was to examine the association between an expert clinician’s impression of symptomatic meniscal tears and subsequent MRI in the context of middle-aged and older adults with knee pain.

**Methods:**

Patients older than 45 were eligible for this IRB-approved substudy if they had knee pain, had not undergone MRI and saw one of two orthopaedic surgeons experienced in the diagnosis of meniscal tear. The surgeon rated their confidence that the patient’s symptoms were due to meniscal tear. The patient subsequently had a 1.5 or 3.0 T MRI within 6 months. We examined the association between presence of meniscal tear on MRI and the surgeon’s confidence that the knee pain was due to meniscal tear using a *χ*^2^ test for trend.

**Results:**

Of 84 eligible patients, 63 % were female, with a mean age of 64 years and a mean BMI of 27. The surgeon was confident that symptoms emanated from a tear among 39 %. The prevalence of meniscal tear on MRI overall was 74 %. Among subjects whose surgeon indicated high confidence that symptoms were due to meniscal tear, the prevalence was 80 % (95 % CI 63–90 %). Similarly, the prevalence was 87 % (95 % CI 62–96 %) among those whose surgeon had medium confidence and 64 % (95 % CI 48–77 %) among those whose surgeon had low confidence (*p* = 0.12).

**Conclusion:**

Meniscal tears were frequently found on MRI even when an expert clinician was confident that a patient’s knee symptoms were not due to a meniscal tear, indicating that providers should use MRI sparingly and cautiously to confirm or rule out the attribution of knee pain to meniscal tear.

**Electronic supplementary material:**

The online version of this article (doi:10.1186/s12891-016-1010-2) contains supplementary material, which is available to authorized users.

## Background

The lateral and medial menisci are fibrocartilaginous, semilunar structures in the tibiofemoral knee compartments that provide load-bearing support and stability. Tears in the meniscus are common among middle-aged and older adults: approximately one-quarter of individuals without and three-fifths of those with radiographic knee osteoarthritis (OA) have a meniscal tear [1, 2]. In fact, over 90 % of persons with symptomatic, advanced radiographic knee OA have a concomitant meniscal tear [3].

Meniscal tears are often implicated as the cause of pain in persons presenting with knee pain and imaging evidence of a tear. The outermost third of the menisci are innervated [4], and a damaged meniscus may itself be the cause of knee pain. However, at least half of persons with meniscal tear visible on magnetic resonance imaging (MRI) experience no knee symptoms [2, 5, 6]. While there is no single clinical history item that definitively indicates the presence of symptomatic meniscal tear, clinical evaluation often focuses on mechanical symptoms such as locking, clicking, popping, localized pain, pain with pivoting and giving way to differentiate symptomatic meniscal tear from other conditions that cause knee pain.

While there is some evidence that these symptoms can help in the diagnosis of symptomatic meniscal tear [7], nonetheless it remains difficult for clinicians to identify a meniscal tear as the primary source of a patient’s symptoms on the basis of history and physical examination. Consequently, clinicians often obtain an MRI to either “confirm” or “rule out” a diagnosis of symptomatic meniscal tear in persons with knee pain [8–10]. However, there has been limited study of the association between presence of tear on MRI in persons with knee pain and whether the tear is the source of pain. Our objective was to evaluate the association between an expert clinician’s impression of the source of knee pain as well as whether meniscal tear was visible on MRI among persons with knee pain seeking clinical care.

## Methods

### Overview

This is a secondary analysis of a parent cross-sectional study designed to evaluate the diagnostic value of individual clinical history and physical examination items in identifying symptomatic meniscal tear. In this analysis, we aimed to evaluate the association between MRI findings and diagnosis of symptomatic meniscal tear.

### Study sample

We recruited participants from the outpatient clinic of two experienced attending orthopedic surgeons (SDM and RJW) between December 2011 and May 2014 at Brigham and Women’s Hospital, an academic medical center in Boston, Massachusetts. All individuals who presented with unilateral knee pain were eligible if they were at least 45 years old, had not seen the surgeon within the preceding year, and had not undergone knee surgery in the past 5 years or total knee arthroplasty at any time.

### Subject evaluation

Participating subjects completed a questionnaire that included the Knee injury and Osteoarthritis Outcomes Score (KOOS, each subscale scored 0–100 with 100 worst) [11]. Following the visit, the surgeon completed a short form indicating his confidence that the patient’s symptoms were due to meniscal tear using a scale from 0 to 100 %, with 0 % indicating that the surgeon was certain that they symptoms *were not* due to meniscal tear and 100 % indicating that the surgeon was certain that the symptoms *were* due to meniscal tear. We divided the surgeon’s confidence that symptoms were due to meniscal tear into three categories: high (67–100 %), medium (34–66 %), and low (0–33 %).

### Imaging acquisition and interpretation

We excluded participants who had a knee MRI available in the hospital medical record system prior to their clinic visit. This exclusion ensured that the surgeons’ assessments were not influenced by MRI findings. Further, we required that participants had MRI performed within 6 months following the baseline visit. To address potential selection bias in the ordering of MRI, individuals were offered the opportunity to participate in a sub-study in which they underwent a research MRI at no cost if their surgeon was not already planning on ordering one. Since we observed that surgeons were more likely to order MRIs for persons in whom they had high confidence in tear, we deliberately oversampled individuals in the low confidence group.

Surgeon-ordered imaging was conducted according to the standard hospital protocol for a clinical knee MRI without intravenous contrast. Using a Siemens 1.5 T, Siemens 3 T, or a General Electric 3 T scanner, the following sequences were obtained: coronal proton density with and without fat saturation, sagittal proton density with and without fat saturation, axial T2 with fat saturation and 3D isotropic proton density acquired sagittally with coronal and axial reformats. The initial read was generally conducted by a radiology resident or fellow. The scan was then read once more by a board certified musculoskeletal radiologist. The research MRIs were obtained using the same protocol and were read by a single board-certified musculoskeletal radiologist.

We abstracted the radiologist’s report of all available MRIs and classified a meniscus as torn if it was macerated or had a signal abnormality that abrogated the meniscal surface. Intrasubstance signal abnormality that did not reach the surface of the meniscus was not classified as a tear. Meniscus that had a post-surgical appearance were not classified as torn.

A non-clinician reader was trained by a musculoskeletal radiologist to read knee X-rays for degenerative changes and graded all available X-rays on the Kellgren-Lawrence scale (KL, graded 0–4 with ≥2 indicating radiographic OA) [12]. The inter-rater reliability for KL grade on a separate sample of radiographs between the reader and radiologist was substantial (weighted kappa 0.72) [13].

### Analysis

We calculated the proportions of individuals with meniscal tear in each confidence category; 95 % confidence intervals were derived using the Wilson Score method [14]. We used a two-tailed *χ*^2^ (Cochran–Armitage) test for trend to investigate a relationship between likelihood of symptomatic meniscal tear diagnosis and presence of tear on MRI [15, 16].

## Results

We recruited 272 individuals with knee pain from December 2011 to May 2014, as depicted in Additional file 1: Figure S1. Of these, 38 patients had an MRI available prior to the clinic visit and were excluded from analysis. Of the remaining 234 persons, 50 (21 %) underwent a clinical MRI scheduled by their physician in the 6 months following the clinic visit. An additional 34 participants (15 %) agreed to undergo a research MRI (Table 1). These 84 subjects with MRIs completed following the visit comprised the study sample.

**Table 1:** Status of MRI following the clinic visit and physician confidence in the diagnosis of symptomatic meniscal tear among persons who did not have imaging available prior to the appointment

Symptomatic Meniscal Tear Confidence	No MRI	MRI ordered by physician	MRI scheduled by study staff	Total
	*N* (%)	*N* (%)	*N* (%)	*N*
High (67–100 %)	18 (38 %)	29 (60 %)	1 (2 %)	48
Medium (34–66 %)	18 (55 %)	9 (27 %)	6 (18 %)	33
Low (0–33 %)	114 (75 %)	12 (8 %)	27 (18 %)	153
Overall (0–100 %)	150 (64 %)	50 (21 %)	34 (15 %)	234

Among the 84 subjects included in the analysis, 53 (63 %) were female, the mean age was 64 years (SD 9), the mean BMI was 27 (SD 6), and the mean KOOS Pain score was 42 (SD 19). Among the 188 patients who either had an MRI before the clinic visit or never had one afterwards (and were therefore excluded from the analysis), 109 (58 %) were female, the mean age was 64 years (SD 10), the mean BMI was 30 (SD 7), and the mean KOOS Pain score was 45 (SD 19).

Among persons who received an MRI, surgeons rated their confidence that the symptoms were due to meniscal tear as high among 30 (36 %) subjects, medium among 15 (18 %) subjects, and low among the remaining 39 (46 %) subjects. There were no meaningful differences in the distributions of the confidence ratings across the two surgeons. Additionally, the proportion of MRIs showing meniscal tear in each of the three groups was similar among participants who had MRI ordered by their physician and those who agreed to undergo a research MRI.

Among the various confidence groups, 24 of 30 subjects (80 %, [95 % CI 63–90 %]) in the high confidence group, 13 of 15 subjects (87 %, [95 % CI 62–96 %]) in the medium confidence group, and 25 of 39 subjects (64 %, [95 % CI 48–77 %) in the low confidence group had a meniscal tear visible on MRI (*p* for trend: 0.12; Table 2). Of the subjects with imaging evidence of meniscal tear, the blinded expert clinician ascribed high confidence of the symptoms being caused by the meniscal tear in 39 %, medium confidence in 21 %, and low confidence in 40 %. The confidence ratings were roughly similar among subjects who did not have meniscal tear documented on MRI.

**Table 2:** Meniscal tears detected on MRIs ordered subsequent to the clinic visit, stratified by surgeon confidence

Symptomatic Meniscal Tear Confidence	Meniscal Tear on MRI	No Meniscal Tear on MRI
	N/Total (%, [95 % CI])	N/Total (%, [95 % CI])
High (67–100 %)	24/30 (80 %, [95 % CI 63–90 %])	6/30 (20 %, [95 % CI 10–37 %])
Medium (34–66 %)	13/15 (87 %, [95 % CI 62–96 %])	2/15 (13 %, [95 % CI 4–38 %])
Low (0–33 %)	25/39 (64 %, [95 % CI 48–77 %])	14/39 (36 %, [95 % CI 23–52 %])
Overall (0–100 %)	62/84 (74 %, [95 % CI 64–82 %])	22/84 (26 %, [95 % CI 18–36 %])

Knee X-rays were available for 78 of 84 (93 %) individuals in our sample who had MRIs. Half had radiographic OA (KL 2–4) and half had normal radiographs with an occasional questionable osteophyte (KL 0–1). The prevalence of MRI-documented meniscal tear was high both among persons with radiographic knee OA (31 of 39, 79 %, [95 % CI 64–89 %]) as well as those without radiographic knee OA (27 of 39, 69 %, [95 % CI 54–81 %]). The limited association between confidence ratings and presence of meniscal tear was observed among both persons with and without radiographic OA (Additional file 2: Table S1).

## Discussion

Among middle-aged and older adults who presented to an outpatient orthopedic clinic for evaluation of knee pain, and who later received an MRI (either ordered by the surgeon or independently by our study staff), about three-quarters had a meniscal tear documented on MRI. This high prevalence of meniscal tear was similar among subjects whom the expert physicians were highly confident had symptomatic meniscal tear (80 % had positive MRI) as well as those for whom the surgeons had medium (87 %) and low levels of confidence (64 %). Meniscal tear prevalence was very high among all patients with radiographic knee OA (79 %) but was also commonly documented among those without radiographic knee OA (69 %).

While the prevalence of meniscal tear on MRI has been studied among asymptomatic individuals and individuals with OA [1–3, 17], there is little published information on the frequency of meniscal tear and the attribution of symptoms among persons with knee symptoms of diverse etiologies. This is an important gap in the literature since patients seen in clinical settings typically have knee pain of diverse causes. Englund et al. ascertained that in a US community setting, approximately half of older persons with frequent knee symptoms had meniscal tear on MRI [2]. Kemp et al. observed in a UK musculoskeletal clinic among patients with knee pain that the prevalence of meniscal tear ranged from 40 % among those with no radiographic evidence of OA to 90 % among patients with advanced radiographic OA [3]. Neither study evaluated the relationship between presence of meniscal tear and likelihood that the patient’s symptoms could be ascribed to a tear. Consistent with our findings, Rathleff et al. noted that about two-thirds of middle-aged individuals with knee pain and suspected symptomatic meniscal tear had a meniscal tear visible on MRI [18].

Several methodologic aspects of our study merit comment. Our study population was drawn from the practices of two academic orthopedic surgeons highly experienced in the diagnosis of knee problems in this age group. Surgeons commonly had access to knee radiographs prior to the clinical encounter, which may have influenced the confidence level of symptom attribution especially if significant degenerative changes were apparent. We used the surgeon’s impression as the gold standard, following practices used in the creation of other classification criteria [19–21], but recognize that the reproducibility of a diagnosis of symptomatic meniscal tear remains largely unstudied [22]. Furthermore, MRIs not systematically obtained, but were instead ordered by the clinician in the normal course of clinical practice. Thus, they could potentially be read and documented by any of the hospital’s diagnostic radiologists, whose intra- and inter-rater reliability are unknown. To address possible selection bias, we obtained research MRIs among an additional set of patients. While our sample of subjects with MRI remained too small to stratify our analysis among the various morphologies of meniscal tear, the age group (mean 67 years) suggests the great majority of the tears were degenerative.

Our findings have important implications for research and practice. Clinical trials for symptomatic meniscal tear that require participants to have knee pain and MRI evidence of meniscal tear with no further entry criteria to refine the clinical syndrome will likely capture a heterogeneous patient population in which many subjects’ knee pain will be unrelated to their meniscal tear. Careful clinical assessment by expert clinicians may reduce this heterogeneity. Additionally, clinicians with relatively little experience evaluating knee pain in middle-aged and older patients may be inclined to obtain MRI to investigate a suspicion of symptomatic meniscal tear as part of a broader differential. Our data suggest that while the great majority of persons presenting with knee pain are likely to have meniscal tear documented on MRI, an orthopedic specialist seeing a tear on MRI would not conclude, on the sole basis of MRI imaging, that the patient’s symptoms are a result of the tear. The symptoms could be caused by a number of other processes, such as bursitis or osteoarthritis. The American College of Radiology suggests that an MRI for non-traumatic knee pain can be considered for persistent pain if X-rays are uninformative and internal derangement is suspected [23]. However, an MRI cannot conclusively determine whether a meniscal tear is symptomatic, and it can be difficult to distinguish between symptomatic meniscal tear and other structural abnormalities. Given the high risk of misattribution, clinicians considering a diagnosis of symptomatic meniscal tear should be cautious of heavily relying on the MRI. Clinicians may instead in this circumstance consider referring the case to a colleague more experienced in differentiating between symptomatic meniscal tear and other causes of knee pain.

## Conclusions

Symptomatic meniscal tear is a common musculoskeletal diagnosis that is generally made on the basis of history, physical examination and imaging. A torn meniscus is often visible on imaging even when expert clinicians are confident on the basis of just history and physical examination that a patient’s knee pain is not due to the torn meniscus. Providers should use therefore MRI sparingly and cautiously to confirm or rule out the attribution of knee pain to meniscal tear.

### Ethics and consent to participate

This study was approved by the institutional review board of Brigham and Women’s Hospital, the Partners Human Research Committee. Participating subjects supplied written informed consent.

### Consent to publish individual patient information

Not applicable.

### Availability of data and materials

The datasets supporting the conclusions of this article are included within the article. If you wish to obtain access for the underlying material please contact the corresponding author to discuss your request in detail.

## Additional files

Additional file 1: Figure S1. Study Flow Diagram. (DOC 170 kb) Additional file 2: Table S1. Meniscal tears detected on MRIs ordered subsequent to the clinic visit, stratified by KL grade and surgeon confidence. (DOC 34 kb)

## Abbreviations

95 % CI: 95 % confidence intervals
BMI: body Mass Index
KL: Kellgren-Lawrence grade
KOSS: knee injury and osteoarthritis outcomes score
MRI: magnetic resonance imaging
OA: osteoarthritis
T: tesla
