# Supplementary figures and images for: Association of MRI findings and expert diagnosis of symptomatic meniscal tear among middle-aged and older adults with knee pain
Source: BMC Musculoskelet Disord. 2016 Apr 11;17:154. doi: 10.1186/s12891-016-1010-2 (PMC4827168; doi:10.1186/s12891-016-1010-2)

**Additional file 1: Figure S1**. Study Flow Diagram


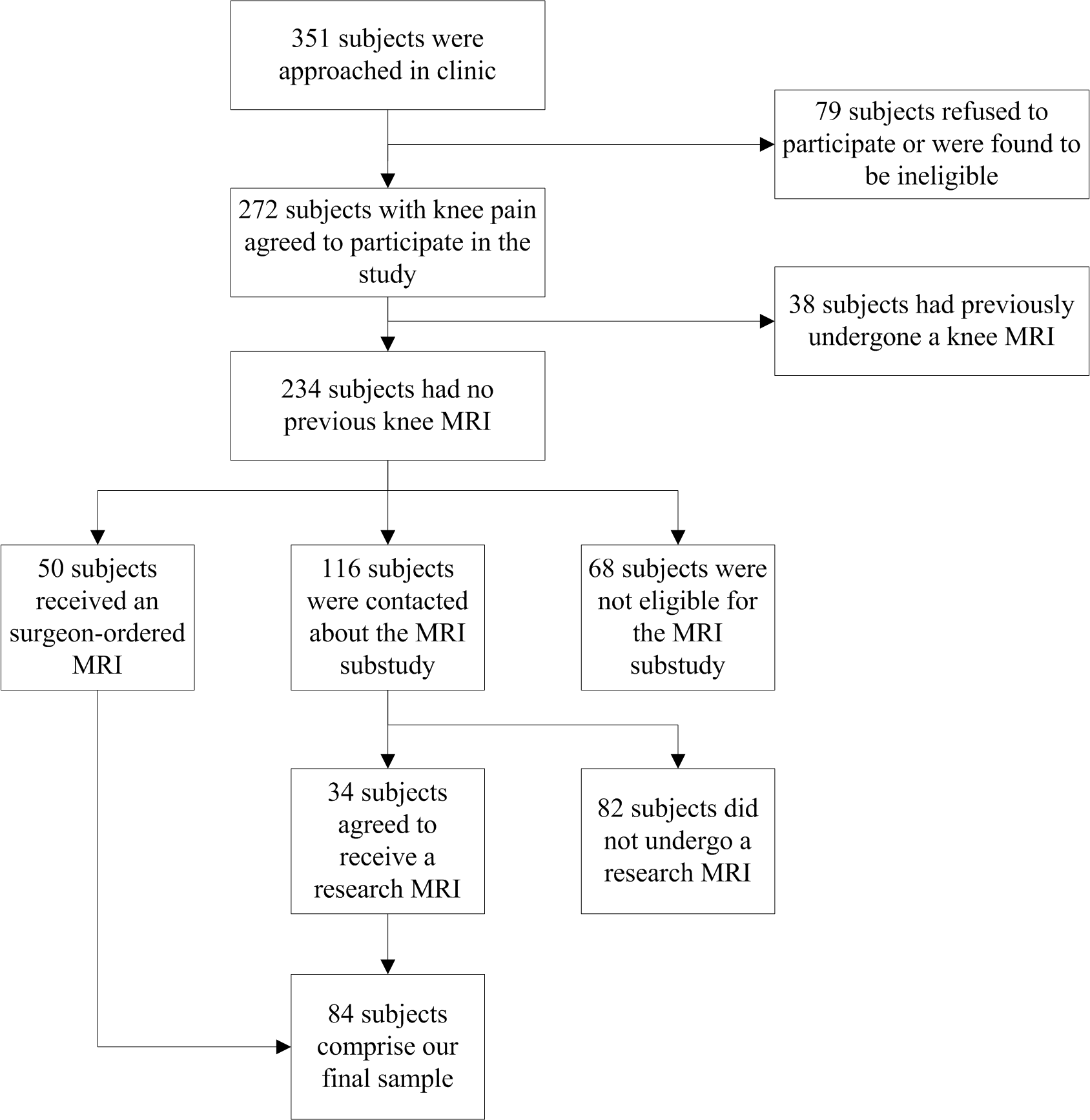

Supplement: Additional file 1: Figure S1. — Study Flow Diagram. (DOC 170 kb) [file 12891_2016_1010_MOESM1_ESM.doc]
