# Supplementary material for: Association of MRI findings and expert diagnosis of symptomatic meniscal tear among middle-aged and older adults with knee pain
Source: BMC Musculoskelet Disord. 2016 Apr 11;17:154. doi: 10.1186/s12891-016-1010-2 (PMC4827168; doi:10.1186/s12891-016-1010-2)
Supplement: Additional file 2: Table S1. — Meniscal tears detected on MRIs ordered subsequent to the clinic visit, stratified by KL grade and surgeon confidence. (DOC 34 kb) [file 12891_2016_1010_MOESM2_ESM.doc]

**Additional file 2: Table S1**. Meniscal tears detected on MRIs ordered subsequent to the clinic visit, stratified by KL grade and surgeon confidence

| **KL Grade** | **Symptomatic Meniscal Tear Confidence** | **Meniscal Tear on MRI** | **No Meniscal Tear on MRI** |
| --- | --- | --- | --- |
|  |  | **N / Total (%, [95% CI])** | **N / Total (%, [95% CI])** |
| X-Ray Unavailable | High (67%–100%) | 1 / 1 (100%, [95% CI 21%–100%]) | 0 / 1 (0%, [95% CI 0%–79%]) |
|  | Medium (34%–66%) | 1 / 1 (100%, [95% CI 21%–100%]) | 0 / 1 (0%, [95% CI 0%–79%]) |
|  | Low (0%–33%) | 2 / 4 (50%, [95% CI 15%–85%]) | 2 / 4 (50%, [95% CI 15%–85%]) |
|  | Overall (0%–100%) | 4 / 6 (67%, [95% CI 30%–90%]) | 2 / 6 (33%, [95% CI 10%–70%]) |
| KL 0-1 | High (67%–100%) | 16 / 21 (76%, [95% CI 55%–89%]) | 5 / 21 (24%, [95% CI 11%–45%]) |
|  | Medium (34%–66%) | 5 / 5 (100%, [95% CI 57%–100%]) | 0 / 5 (0%, [95% CI 0%–43%]) |
|  | Low (0%–33%) | 6 / 13 (46%, [95% CI 23%–71%]) | 7 / 13 (54%, [95% CI 29%–77%]) |
|  | Overall (0%–100%) | 27 / 39 (69%, [95% CI 54%–81%]) | 12 / 39 (31%, [95% CI 19%–46%]) |
| KL 2-4 | High (67%–100%) | 7 / 8 (88%, [95% CI 53%–98%]) | 1 / 8 (13%, [95% CI 2%–47%]) |
|  | Medium (34%–66%) | 7 / 9 (78%, [95% CI 45%–94%]) | 2 / 9 (22%, [95% CI 6%–55%]) |
|  | Low (0%–33%) | 17 / 22 (77%, [95% CI 57%–90%]) | 5 / 22 (23%, [95% CI 10%–43%]) |
|  | Overall (0%–100%) | 31 / 39 (79%, [95% CI 64%–89%]) | 8 / 39 (21%, [95% CI 11%–36%]) |
